# Release and Cytocompatibility Study of New Hybrid Materials Based on Ferulic Acid for Biomedical Use

**DOI:** 10.3390/ijms26178450

**Published:** 2025-08-30

**Authors:** Federico Barrino, Federica Giuliano, Clelia Dispenza

**Affiliations:** Department of Engineering, University of Palermo, Viale delle Scienze 6, 90128 Palermo, Italy; federica.giuliano02@unipa.it (F.G.); clelia.dispenza@unipa.it (C.D.)

**Keywords:** hybrid material, sol–gel, natural drug, release kinetics

## Abstract

In recent years, research into the synthesis of innovative biomaterials for prosthetic applications has been increasingly growing. In particular, there is a demand for biomaterials with an excellent biocompatibility that can interact with biological fluids. This study involved the development of new silica (SiO_2_)-based composite materials using the sol–gel technique and functionalization with ferulic acid (FA), a natural phenolic compound renowned for its biological properties. The synthesis involved controlling the hydrolysis and condensation of tetraethyl orthosilicate (TEOS) in acidic and alcoholic environments to incorporate ferulic acid into the sol phase matrix at different weight compositions (5, 10, 15, and 20 wt%). Fourier transform infrared spectroscopy analyses (FTIR) confirmed the successful incorporation of the bioactive compound, and in vitro tests revealed a good cytocompatibility and controlled ferulic acid release over time. These results demonstrate that the developed material shows promise as a bioactive coating for orthopedic prostheses, improving bone integration and reducing undesirable post-operative phenomena.

## 1. Introduction

Regenerative medicine is one of the most dynamic and promising fields of contemporary biomedical research. It aims to restore the functionality of damaged tissues and organs through strategies that stimulate the body’s natural repair processes [1]. In this context, tissue engineering has assumed a key role, focusing on the design of advanced biomaterials capable of supporting and guiding cellular regeneration. These materials must possess a series of fundamental requirements: biocompatibility, controlled biodegradability, adequate mechanical support, and, increasingly, specific biological activity [2].

Among the numerous biomaterials studied for regenerative applications, silicon dioxide (SiO_2_)-based materials have stood out for their excellent chemical stability, compatibility with the physiological environment, and ability to be easily functionalized. In particular, the sol–gel technique has enabled the development of high-performance organic–inorganic hybrid materials [3,4]. This synthesis method, conducted at low temperatures and in a controlled environment, is based on hydrolysis and condensation reactions of organosilicon precursors such as tetraethyl orthosilicate (TEOS) that lead to the formation of a three-dimensional network of amorphous silica [5]. One of the most interesting aspects of sol–gel chemistry is the ability to incorporate bioactive molecules within the inorganic matrix, preserving their functionality and controlling their release over time [6,7].

In this context, the introduction of natural compounds with therapeutic value into sol–gel materials represents an effective strategy for obtaining multifunctional biomaterials. Indeed, these materials are capable not only of providing structural support, but also of exerting a targeted biological action. A prime example is ferulic acid (FA), a plant-derived phenolic molecule known for its antioxidant, anti-inflammatory, and antibacterial properties [8]. These characteristics make it particularly attractive for biomedical applications, as it can counteract oxidative stress and inflammation, two conditions often associated with pathological and tissue repair processes. However, to ensure its long-term therapeutic efficacy, it is essential that ferulic acid be stably and efficiently incorporated into the biomaterial matrix, without compromising its bioactivity [9,10].

The use of the sol–gel technique not only protects ferulic acid from premature degradation, but also modulates its concentration within the material, paving the way for a systematic study of dose-dependent effects. In particular, varying the weight percentage of FA can significantly influence the structural, chemical, and biological properties of the final material [11,12]. At lower concentrations, ferulic acid, like most herbal drugs, may behave primarily as an antioxidant, while at higher concentrations, it may also exert a pro-inflammatory or cytotoxic effect, necessitating a careful evaluation of its interaction with cells and tissues.

This study aimed to synthesize and characterize SiO_2_-based hybrid biomaterials containing ferulic acid at varying concentrations (5, 10, 15, and 20% by weight), with the aim of understanding how the FA content influences the chemical, physical, and biological properties of the resulting materials. Specifically, the formation of interactions between the components was examined using FTIR-ATR spectroscopy, including the potential release of the herbal drug over time in three different media (H_2_O, PBS, and SBF), the gene expression, and the cytocompatibility, to assess the biological compatibility of the developed materials regarding their application in tissue regeneration, for example, as scaffolds for cell growth or as functionalized coatings for implantable devices.

This integrated approach, which combines the chemistry of sol–gel materials with the pharmacology of natural compounds, aims to contribute to the development of new therapeutic solutions based on smart biomaterials, capable of providing not only mechanical support, but also active and controlled therapeutic activity over time [13]. The results obtained may provide useful insights for optimizing the composition of biomaterials and their subsequent clinical application, particularly in the treatment of tissue lesions, chronic wounds, and implantology [14,15,16].

## 2. Results

### 2.1. Sol–Gel Synthesis

Hybrid biomaterials based on SiO_2_ containing ferulic acid (FA) in increasing concentrations (5%, 10%, 15%, and 20% by weight) were successfully synthesized using the sol–gel process (Table 1). This synthesis method, conducted at low temperatures and in a controlled environment, is based on hydrolysis and condensation reactions of organosilicon precursors such as tetraethyl orthosilicate (TEOS) that lead to the formation of a three-dimensional network of amorphous silica. All samples exhibited a good workability and physical stability at room temperature, without any evident phenomena of phase separation or the macroscopic crystallization of the organic component. The synthesis produced transparent and homogeneous gels with a controlled gelling time of about 48–72 h at room temperature. The gelled materials exhibited a gradual color change with an increasing weight percentage of the incorporated ferulic acid. Pure SiO_2_ is a colorless material, while SiO_2_/FA5_wt_% had a yellowish color, SiO_2_/FA10_wt_% was orange, SiO_2_/FA15_wt_% was red, and SiO_2_/FA20_wt_% was burgundy.

### 2.2. Interactions Between SiO_2_ and SiO_2_/FA

**Figure 1:**
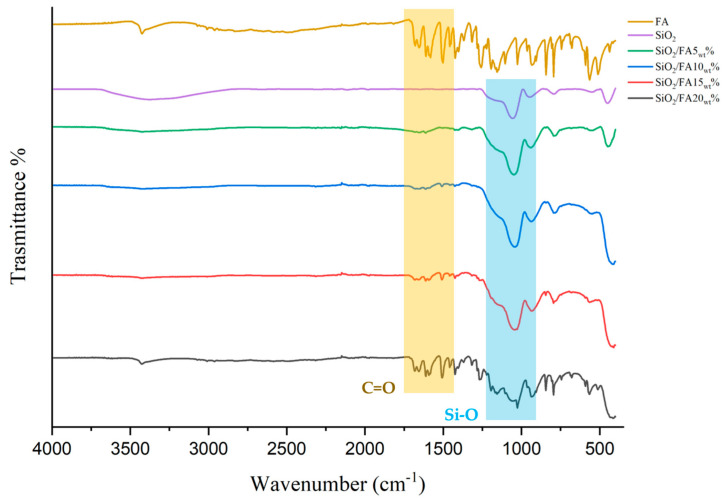
FTIR-ATR spectra of pure drug (FA), silica matrix (SiO_2_), and synthesized hybrid materials (SiO_2_/FA5, 10, 15 ad 20_wt_%).

The FTIR analysis confirmed the effective incorporation of ferulic acid into the silica matrix (Figure 1). Typical Si–O–Si asymmetric stretching signals were observed in all samples around 1050 cm^−1^ and 800 cm^−1^; furthermore, the silicon spectrum features Si–OH at 960 cm^−1^ and H–O–H bending at 1630 cm^−1^. The ferulic acid spectrum shows O–H stretching at 3400 cm^−1^, carboxylic C=O at ~1680–1700 cm^−1^, and aromatic C=C ~1600–1510 cm^−1^. The spectra of the hybrid materials show bands along with the aromatic ring peak (around 1600 cm^2^) and the carbonyl group (around 1700 cm^2^). The relative intensity of these bands increased proportionally to the weight percentage of fatty acids, suggesting the increasing and stable incorporation of the active ingredient into the inorganic network. Specifically, 5% exhibit weak interactions; 10% exhibit more evident H-bond interactions; 15% exhibit an increase in C=O signals and possible Si–O–C bonds; and finally, 20% exhibit a spectrum dominated by organic signals, suggesting possible saturation interactions with silica.

### 2.3. Kinetics Release

Four different hybrid materials were synthesized by incorporating increasing weight percentages of ferulic acid (5, 10, 15, and 20 wt%) into the SiO_2_ system, and their release behavior was studied over a 24 h period in three different media. Two initial loading concentrations, 0.5 mg/mL (blue curves) and 1 mg/mL (orange curves), were used to investigate the effect of the FA dosage on release behavior.

**Figure 2:**
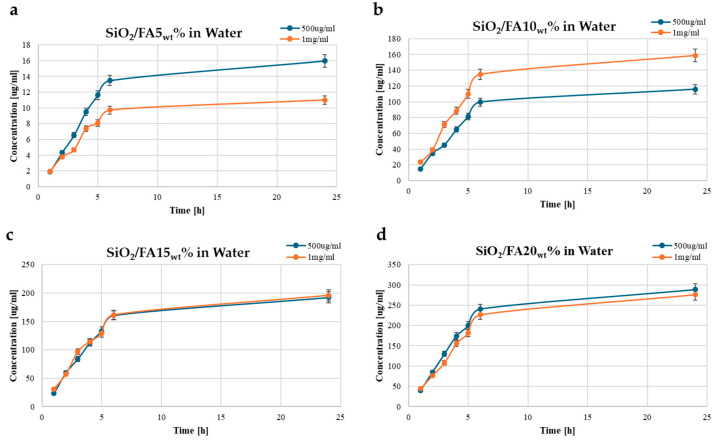
Time-dependent drug release plots for (**a**) SiO_2_/FA5_wt_%, (**b**) SiO_2_/FA10_wt_%, (**c**) SiO_2_/FA15_wt_%, and (**d**) SiO_2_/FA20_wt_% in ultrapure water medium. Concentration is expressed in μg/mL.

Figure 2 presents the release profiles of ferulic acid (FA) from SiO_2_-based hybrid materials in ultrapure water. Figure 2a shows the release from SiO_2_/FA5_wt_%, where the 0.5 mg/mL sample achieved a final concentration close to 16 mg/L, while the 1 mg/mL formulation plateaued at a lower concentration (~11 mg/L). The release was rapid in the first few hours, with a gradual increase up to 24 h. In Figure 2b, SiO_2_/FA10_wt_% exhibits higher cumulative release values, with both concentrations displaying similar kinetic trends, but differing final concentrations (~160 mg/L for 1 mg/mL vs. ~120 mg/L for 0.5 mg/mL), indicating an increased FA availability with higher loading.

Figure 2c illustrates the release from SiO_2_/FA15_wt_%, where both initial concentrations reached similar final values (~190–200 mg/L), suggesting a saturation point in the release medium. Figure 2d shows SiO_2_/FA20_wt_%, which demonstrated the highest release, reaching up to ~290 mg/L for the 0.5 mg/mL concentration and slightly lower (~270–280 mg/L) for the 1 mg/mL, likely due to diffusion limitations or solubility constraints. These results reveal a direct correlation between the FA loading percentage and the release amount up to 20 wt%, with a rapid initial release within the first 5 h and a plateauing trend thereafter. This controlled release behavior supports the potential of these hybrid materials in sustained drug delivery applications.

**Figure 3:**
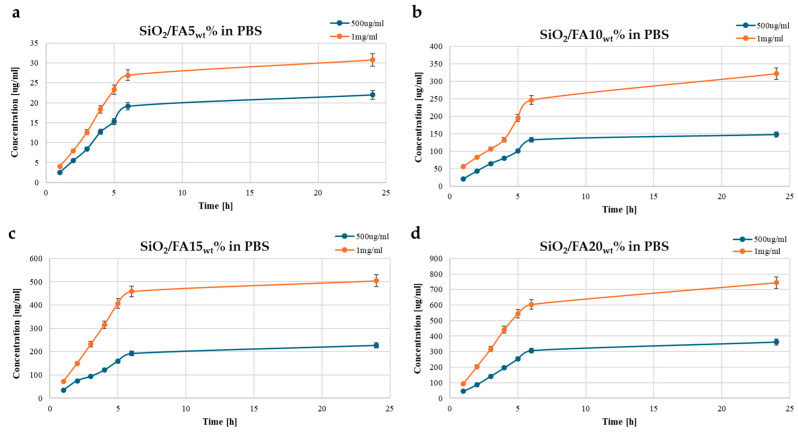
Time-dependent drug release plots for (**a**) SiO_2_/FA5_wt_%, (**b**) SiO_2_/FA10_wt_%, (**c**) SiO_2_/FA15_wt_%, and (**d**) SiO_2_/FA20_wt_% in PBS medium. Concentration is expressed in μg/mL.

Figure 3 illustrates the release profiles of ferulic acid (FA) from SiO_2_-based hybrid materials into phosphate-buffered saline (PBS). Figure 3a shows the release from SiO_2_/FA5_wt_%, where both concentrations exhibited a fast initial release within the first 5 h, followed by a gradual plateau.

The 1 mg/mL system reached a higher final concentration (~31 mg/L) compared to the 0.5 mg/mL sample (~22 mg/L), suggesting a loading-dependent release profile. In Figure 3b, the SiO_2_/FA10_wt_% shows a more pronounced trend, with the 1 mg/mL curve reaching ~322 mg/L and the 0.5 mg/mL curve stabilizing at ~150 mg/L. Figure 3c shows the release behavior of the SiO_2_/FA15_wt_% system, where the 1 mg/mL sample released roughly half of its total load, indicating a significantly higher release compared to the 0.5 mg/mL sample (~230 mg/L, slightly less than half), which also exhibited moderately slower release kinetics. Similarly, in Figure 3d, SiO_2_/FA20_wt_% shows the highest overall release, with the 1 mg/mL system reaching ~750 mg/L and the 0.5 mg/mL curve levelling off near 360 mg/L. These results demonstrate a clear trend of increased FA release with higher FA loading in the SiO_2_ matrix. The sharp release in the early stages (within 5 h) followed by a plateau indicates a typical biphasic release profile—an initial burst followed by sustained diffusion. Compared to the release profiles in ultrapure water (Figure 3), the higher ionic strength and buffering capacity of PBS appear to facilitate a greater total release of FA, highlighting the potential of these hybrid materials for physiological conditions and controlled drug delivery applications.

**Figure 4:**
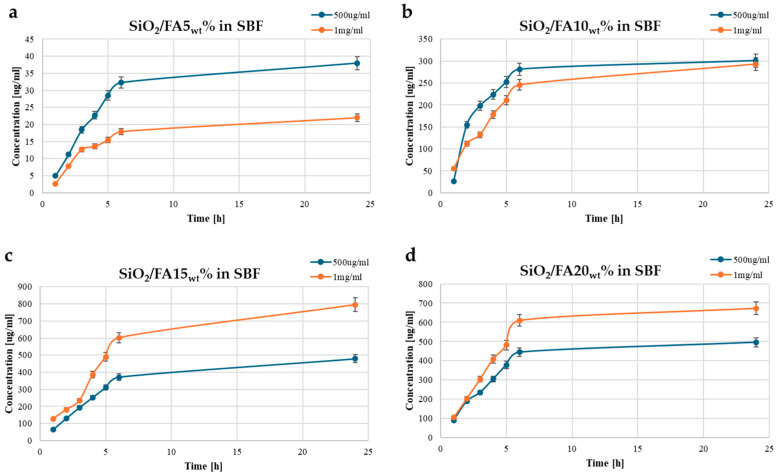
Time-dependent drug release plots for (**a**) SiO_2_/FA5_wt_%, (**b**) SiO_2_/FA10_wt_%, (**c**) SiO_2_/FA15_wt_%, and (**d**) SiO_2_/FA20_wt_% in SBF medium. Concentration is expressed in μg/mL.

Figure 4 illustrates the time-dependent release profiles of ferulic acid (FA) from a silica (SiO_2_) matrix in simulated body fluid (SBF). Figure 4a shows the release profile of SiO_2_/FA5_wt_%, where both concentrations exhibited a rapid release within the first 5 h, followed by a plateau phase, indicating the saturation of the medium. Figure 4b represents SiO_2_/FA10_wt_%, where the release rate increased compared to the 5 wt% sample, suggesting a higher diffusion gradient and possibly greater FA availability. In Figure 4c, the SiO_2_/FA15_wt_% system demonstrates a significant rise in the total release, with the 1 mg/mL sample reaching ~800 mg/L, highlighting the enhanced release efficiency at this loading. Finally, Figure 4d shows the release behavior for SiO_2_/FA20_wt_%, which demonstrated high release levels comparable to the 15 wt% system, but with slightly lower overall amounts, suggesting that the system may already be approaching its saturation point at 15 wt%. Increasing the FA loading beyond this threshold does not appear to significantly boost the total release, and instead may only slow down the release kinetics. Overall, the data indicate that increasing the FA content in the SiO_2_ matrix enhances the release amount, which is particularly evident between 10 wt% and 15 wt%, with diminishing returns at 20 wt%.

### 2.4. Biocompatibility

#### 2.4.1. Cytocompatibility

**Figure 5:**
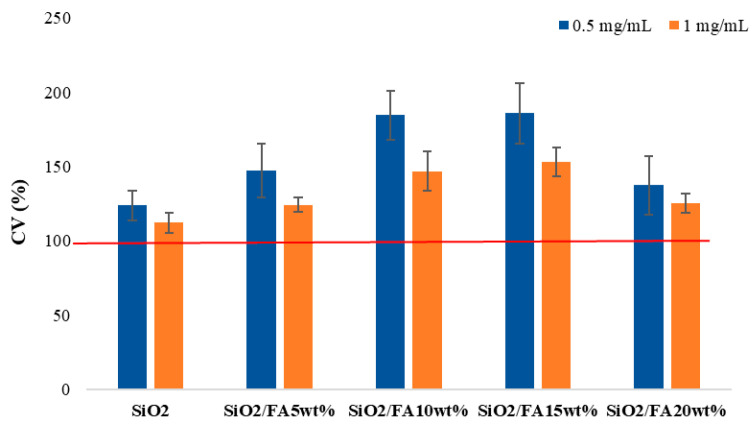
Cytocompatibility of the synthesized materials compared with the CTR (SiO_2_) at two different dosages (0.5 and 1.0 mg/mL).

As displayed in Figure 5, none of the materials resulted in cytotoxicity for the cells. An independent sample *t*-test, with *p* < 0.05, proved that a significant increase in cell viability compared to CTR was found for all the samples except for SiO_2_. In general, 0.5 mg/mL proved to have a greater efficacy with respect to the 1 mg/mL dose. However, at both tested concentrations, SiO_2_/FA10_wt_% and SiO_2_/FA_wt_15% had a very similar effect. In fact, at 0.5 mg/mL, the cell viability was 184% and 185%, respectively, for SiO_2_/FA_wt_10% and SiO_2_/FA_wt_15%, while at 1 mg/mL, it was 143% and 153% for SiO_2_/FA_wt_10% and SiO_2_/FA_wt_15%.

#### 2.4.2. Gene Expression

**Figure 6:**
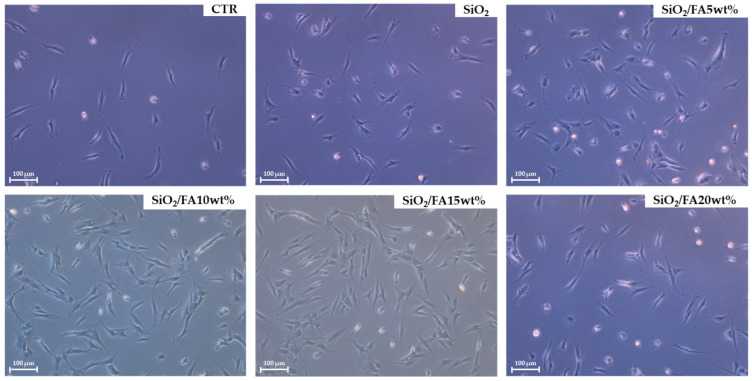
Representative pictures of cells after treatments; scale bar: 100 μm, 10× magnification.

The cells were found to be viable and did not show any morphological changes after treatment (Figure 6). Consequently, RNA extraction was performed.

**Figure 7:**
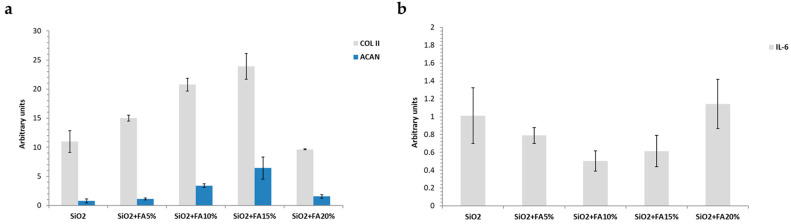
Gene expression of (**a**) COL II and ACAN compared to CTR and (**b**) IL-6 compared to CTR.

Figure 7a showed that all the materials sustained the gene expression of COL II and ACAN compared to CTR. In particular, SiO_2_/FA_wt_10% and SiO_2_/FA_wt_15% were the most performant; in fact, in the presence of SiO_2_/FA_wt_10%, there was a 20.7- and 3.4-fold upregulation of COL II and ACAN, respectively, while SiO_2_/FA_wt_15% increased these biomarkers by 23.8- and 6.4-fold. In addition, each materials reduced IL-6 compared to CTR; the best results were obtained again by SiO_2_/FA_wt_10% and SiO_2_/FA_wt_15% with a very similar trend. Finally, SiO_2_/FA_wt_20% was not very effective at reducing the IL-6 gene expression (Figure 7b).

## 3. Discussion

Advanced silica-based biomaterials for regenerative medicine applications represent one of the most promising strategies for developing multifunctional therapeutic systems capable of supporting cell growth while simultaneously delivering bioactive compounds [17]. In this study, organic–inorganic hybrid materials were synthesized using the sol–gel technique, starting from silane precursors (TEOS) and incorporating varying weight percentages of ferulic acid (FA) with the aim of obtaining bioactive and cytocompatible systems suitable for tissue regeneration applications.

The data obtained by FTIR-ATR spectroscopy confirmed the formation of the silica network through the classic Si–O–Si bond stretching bands around 1050 cm^−1^ [18,19]. The presence of ferulic acid was successfully detected in FA-containing materials, thanks to the observation of characteristic bands associated with aromatic (1600 cm^−1^) and carbonyl (≈1700 cm^−1^) functional groups [20]. As the FA incorporation percentage increased, these bands progressively intensified, which was consistent with increasing the integration of the bioactive compound into the inorganic matrix [21,22].

However, in samples containing 20% FA, some bands were more nuanced and broadened, indicating possible interactions between FA molecules or partial self-association, which could indicate the saturation of the silica network in the ordered incorporation capacity of the compound. No obvious signs of the chemical degradation of the FA were observed during the synthesis, confirming that the temperature and pH conditions adopted in the sol–gel process are compatible with the stability of the molecule.

The FA release profile was evaluated using two different concentrations (0.5 and 1 mg/mL) under three different experimental conditions (H_2_O, PBS, and SBF) to simulate different clinical contexts such as inflammatory environments or chronic wounds [23,24,25]. The release profiles of ferulic acid from the SiO_2_-based hybrid matrices demonstrated a strong dependence on both FA loading and the release medium. In all three environments, the release followed a characteristic trend of an initial rapid burst within the first five hours, followed by a plateau indicative of diffusion-controlled behavior. Increasing the FA loading from 5 wt% to 15 wt% consistently enhanced the total release across all media, while a further increase to 20 wt% yielded only marginal gains, suggesting a saturation point in the matrix [26,27]. Importantly, the release medium significantly influenced the overall quantity of FA delivered, with the highest release observed in SBF, followed by PBS, and the lowest in ultrapure water. These results highlight the interplay between the material’s composition and the ionic environment, confirming the potential of SiO_2_-based hybrid matrices for tailored and sustained drug delivery across a range of physiological conditions.

In any case, the presence of residual FA at extended times demonstrates that, in all formulations, a portion of the active ingredient remains trapped in the silica structure for sufficient time to ensure sustained release, a particularly relevant aspect for applications in slowly regenerating tissues.

The cytocompatibility of the biomaterials was assessed on chondrocyte cell cultures using MTT assays and morphological observations. All the samples with FA up to 15% showed a good cellular tolerance after 72 h of incubation. The cells maintained an elongated and adherent morphology, indicating good interaction with the substrate. In the samples with 20% FA, however, a slight reduction in viability was observed, suggesting a possible threshold concentration beyond which local FA release may become cytotoxic for some sensitive cell types [28,29].

These results indicate that there is an optimal range of FA incorporation by weight (between 10 and 15%) in which it is possible to effectively combine bioactivity and biocompatibility, while avoiding unwanted side effects. This aspect is crucial for clinical use, where direct interaction between the material and host cells is a key element for successful tissue regeneration [30,31].

A gene expression analysis provided further information on the bioactive potential of the materials. In particular, biomaterials containing 10% or 15% FA induced an increased expression of pro-regenerative markers, such as COL II and ACAN, compared to the control (SiO_2_ without FA). These data suggest that FA released in a controlled manner from the matrix is able to positively modulate the cellular environment, stimulating fundamental processes such as extracellular matrix deposition and neoangiogenesis [32,33].

Conversely, samples with 20% FA showed a tendency to upregulate genes related to oxidative stress or apoptosis, such as IL-6, indicating that excessively high concentrations can trigger undesirable cellular responses. Materials with 5% FA, while well-tolerated, showed less gene activity, suggesting that, at low concentrations, FA bioactivity may not be sufficient for a significant therapeutic effect.

Overall, the data obtained demonstrate that SiO_2_-based biomaterials synthesized via sol–gel and functionalized with ferulic acid represent a versatile and promising platform for regenerative medicine. Different FA weight percentages significantly influence not only the chemical structure and release kinetics of the material, but also its interaction with cells and the associated gene response [34].

Of all the formulations tested, those with 10% or 15% ferulic acid proved to be the most balanced in terms of their bioactivity, stability, and cytocompatibility, demonstrating good release control, the stimulation of regenerative gene expression, and the absence of significant cytotoxic effects. These results suggest that these compositions may be excellent candidates for the development of bioactive scaffolds, implant coatings, or matrices for the local release of anti-inflammatory materials in clinical settings related to tissue repair.

Further in vivo studies and long-term functional testing will be necessary to confirm the therapeutic potential of these systems in real clinical applications, but the chemical, physical, and biological basis demonstrated in this work offers a solid platform for future developments.

## 4. Materials and Methods

### 4.1. Synthesis by Sol–Gel Technique

The synthesis of silicon dioxide (SiO_2_)-based biomaterials was carried out using the sol–gel method in an aqueous–alcoholic environment, starting from the precursor TEOS (Sigma-Aldrich, St. Louis, MO, USA). This process proved particularly suitable for the incorporation of bioactive compounds such as ferulic acid (≥99%, Sigma-Aldrich), allowing for the formation of organic–inorganic hybrid materials under mild conditions and at low temperatures, thus preserving the functionality of the active ingredient.

In a first step, TEOS was dissolved in absolute ethanol (99.8%, Sigma-Aldrich) under magnetic stirring. At the same time, an acidified aqueous solution was prepared to activate the hydrolysis and subsequent condensation reactions of the silane precursor. Distilled water and nitric acid (≥65%, Sigma-Aldrich) were slowly added to the alcoholic TEOS solution, maintaining a molar ratio of TEOS/HNO_3_ = 1.6; EtOH/TEOS = 6.2; H_2_O/TEOS = 6. The mixture was stirred for at least 30 min until a clear, homogeneous sol was obtained [35].

A solution of ferulic acid was then prepared in heated ethanol to promote complete solubilization. Depending on the desired percentage of FA in the final material, amounts corresponding to 5%, 10%, 15%, and 20% by weight of the total dry mass of the system were weighed. The FA solution was then slowly added to the silica sol under constant stirring, ensuring the homogeneous distribution of the compound within the developing inorganic matrix. Stirring continued for at least 1 h at room temperature to ensure FA integration into the developing silica network.

**Figure 8:**
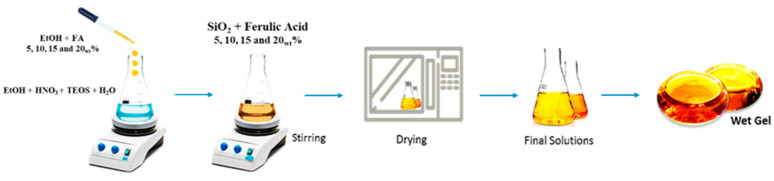
Sol–gel procedure used to obtain hybrid materials.

The resulting mixture was left to stand at room temperature to promote the gelation process, which occurred within 48–72 h, depending on the composition. The flow chart of sol–gel synthesis from solution preparation to gel formation is shown in Figure 8. After gelation, the samples were dried at 30–40 °C in a static oven to gradually remove residual solvents and obtain powders according to the experimental needs. The heat treatment was conducted with particular care to avoid compromising the thermal stability and chemical integrity of the ferulic acid.

### 4.2. FTR-ATR Analysis

ATR/FTIR spectroscopy was used in this investigation to evaluate the chemical interactions between the materials. A Perkin-Elmer FT-IR/NIR Spectrum 400 spectrophotometer equipped with an AIM-8800 infrared microscope (Shimadzu, Tokyo, Japan) was used to obtain the spectra. This procedure made use of an integrated 3 mm diameter Ge attenuated total reflectance (ATR) semicircular prism. The spectra were acquired at a resolution of 4 cm^−1^ (64 scan) and an incidence angle of 30°, with a range of 340–4340 cm^−1^.

### 4.3. Drug Release

The hybrid SiO_2_-based materials were finely ground using an agate mortar and pestle to obtain a homogeneous powder with a reduced particle size, ensuring reproducible surface area exposure during the release experiments. For the release studies, approximately 15 mL of each of the three media was used to suspend the materials: ultrapure water (0.055 µS/cm, pH of 6.97), phosphate-buffered saline (PBS, 1×, pH of 7.4), and simulated body fluid (SBF, pH of 7.4). These media were selected to simulate different physiological environments and evaluate their influence on ferulic acid (FA) release.

The release of FA from the hybrid matrices was monitored over time to assess the drug delivery performance. A quantitative analysis was carried out using a Jasco V-670 UV–vis spectrophotometer (JASCO EUROPE s.r.l., Cremella, Italy). Absorbance measurements were performed at λ = 286 nm, corresponding to the characteristic maximum absorbance of ferulic acid in the tested media.

For each time point, aliquots were collected from the suspension and transferred to quartz cuvettes for the spectroscopic analysis. The instrument was operated over a wavelength range of 200–800 nm, with a scan speed of 100 nm/min and a bandwidth of 1 nm. All measurements were conducted at room temperature (24 ± 2 °C). Baseline corrections were applied using the corresponding blank medium, and all the experiments were performed in triplicate to ensure reproducibility.

#### 4.3.1. PBS Preparation

Phosphate-buffered saline (PBS 1×, pH 7.4) is a commonly used isotonic buffer in biological and pharmaceutical research, mimicking physiological ionic strength and pH. The solution was prepared by dissolving appropriate amounts of monopotassium phosphate (Sigma-Aldrich), sodium chloride (Sigma-Aldrich), potassium chloride (Sigma-Aldrich), and disodium phosphate (Sigma-Aldrich) in ultrapure water, followed by a pH adjustment to 7.4.

#### 4.3.2. SBF Preparation

Simulated body fluid (SBF) was prepared according to the protocol developed by Kokubo and Takadama [36,37], designed to closely replicate the inorganic ion concentrations of human plasma. The following reagents were sequentially dissolved in ultrapure water under constant stirring at room temperature: NaCl, NaHCO_3_, KCl, K_2_HPO_4_·3H_2_O, MgCl_2_·6H_2_O, CaCl_2_, Na_2_SO_4_, and 4-(2-hydroxyethyl)-1-piperazine methanesulfonic acid (HEPES). The pH was adjusted to 7.4 using 1 M HCl.

#### 4.3.3. Calibration Curve

To ensure the accurate quantification of FA in each release medium, separate calibration curves were established in ultrapure water, phosphate-buffered saline (PBS), and simulated body fluid (SBF). Standard solutions of ferulic acid were prepared in each medium at known concentrations ranging from 1 to 15 μg/mL. The absorbance values were plotted against the corresponding concentrations, and a linear regression analysis was performed. Medium-specific calibration was essential to account for matrix effects, particularly in PBS and SBF, where ionic components can shift the baseline absorbance and influence FA’s spectral behavior.

The calibration curve of ferulic acid in ultrapure water has the following equation:

y = 153.62x + 0.0091    (R^2^ > 0.998) (1)

The calibration curve of ferulic acid in PBS has the following equation:

y = 106.47x − 0.123    (R^2^ > 0.998) (2)

The calibration curve of ferulic acid in SBF has the following equation:

y = 61.779x + 0.0465    (R^2^ > 0.998) (3)

where x is the concentration of FA (mg/mL) and y is the absorbance at 286 nm.

### 4.4. Cytotoxicity Assay

The eventual cytotoxicity of the product materials was evaluated by an MTT (3-(4,5-dimethyl-2-thiazolyl)-2,5-diphenyl-2H-tetrazolium bromide) assay on a human immortalized chondrocyte cell line. The cells were grown in the following culture medium: Dulbecco’s modified Eagle medium supplemented with 10% fetal bovine serum, 50.0 U/mL of penicillin, and 100.0 μg/mL of streptomycin, at 37 °C in a humidified atmosphere with 5% CO_2_. Each material was tested here at concentrations of 0.5 and 1 mg/mL (*w*/*v*). In detail, 2 × 10^5^ cells/well were seeded in a standard 24-well plate and the cytotoxicity test was performed after 48 h of incubation. To achieve this, 200 μL of MTT solution (0.5 mg/mL) was added to the cells after 3 h, DMSO was used to solubilize the salts of formazan and the relative optical densities were measured at 570 nm by a microplate reader (Tecan, Männedorf, Switzerland). The cellular viability was calculated by comparing the absorbance of each sample to untreated cells (CTR) as a percentage. The cell line used was as follows: CHON-001–CRL-2846. “CHON-001” is a chondrocyte cell line with a fibroblast-like morphology that was isolated in 2001 from the cartilage of an 18-week-old, normal female donor. This cell line was deposited by ATCC and can be used in drug development research.

### 4.5. Real-Time Quantitative PCR

In order to mimic osteoarthritis conditions, the chondrocytes were pre-insulted with IL-1β (5 ng/mL), as previously reported by Calamia et al. 2012 [38]. Then, each powder material was dissolved in culture medium at a 0.5 mg/mL concentration, which was chosen because it gave the best results in terms of cytotoxicity. Thus, 5 × 10^5^ cells/well were seeded in a standard 24-well plate and cultivated in vitro on biomaterials for 48 h. Real-time PCR analyses were performed to evaluate the gene expression of type II collagen, cartilage, pro-inflammatory-specific markers (Aggrecan (ACAN), and interleukin 6 (IL-6)). Intracellular RNA was isolated by Trizol (Invitrogen, Waltham, MA, USA) following the manufacturer’s instructions and 1000 ng of each RNA sample was reverse-transcribed using a cDNA synthesis kit (Promega, Madison, WI, USA). The quantitative PCR analyses were carried out using SYBR Green Master Mix (Roche, Basel, Switzerland) and a LightCycler 480 instrument (Roche). The results were analyzed using the LightCycler 480 software version 1.5.0.

## 5. Conclusions

Synthesizing hybrid biomaterials based on silica and ferulic acid using the sol–gel technique results in transparent, homogeneous gels with a controlled gelation time of around 48–72 h at room temperature. An FTIR analysis revealed characteristic siloxane peaks at around 1100 cm^−1^, confirming the formation of the silica network (Si–O–Si). The presence of ferulic acid was indicated by aromatic and carboxylic peaks at around 1600 and 1700 cm^−1^, respectively, demonstrating its effective incorporation into the inorganic matrix. In vitro release tests revealed sustained ferulic acid release kinetics over 24 h, comprising an initial rapid phase within the first 6 h followed by a slower, more consistent phase. This profile suggests the potential for prolonged biological effects over time. Finally, cytocompatibility tests revealed an excellent compatibility of the material, with cell proliferation similar to or greater than that of the control, indicating an absence of cytotoxic effects due to the matrix or ferulic acid release. In conclusion, the biomaterials that performed best in the initial characterization phase were SiO_2_/FA10_wt_% and SiO_2_/FA15_wt_%.

## Figures and Tables

**Table 1:** Products obtained from sol–gel synthesis.

Label	System Composition	
	Inorganic Matrix SiO_2_ (wt%)	Organic Matrix FA (wt%)
SiO_2_	100	---
SiO_2_/FA5_wt_%	95	5
SiO_2_/FA10_wt_%	90	10
SiO_2_/FA15_wt_%	85	15
SiO_2_/FA20_wt_%	80	20

## Data Availability

The original contributions presented in this study are included in the article. Further inquiries can be directed to the corresponding author.

## Abbreviations

The following abbreviations are used in this manuscript:

FA	Ferulic acid
TEOS	Tetraethyl orthosilicate
FTIR	Fourier transform infrared spectroscopy
ATR	Attenuated total reflectance
MTT	(3-(4,5-Dimethyl-2-thiazolyl)-2,5-diphenyl-2H-tetrazolium bromide)
ACAN	Aggrecan
IL-6	Interleukin 6
COL II	Type II collagen
